# Predictive value of preoperative circulating tumor cells combined with hematological indexes for liver metastasis after radical resection of colorectal cancer

**DOI:** 10.1097/MD.0000000000041264

**Published:** 2025-01-10

**Authors:** Tianyi Zhu, Yunsong Li, Rui Li, Jingjing Zhang, Wentao Zhang

**Affiliations:** a Department of Clinical Laboratory, The Third Hospital of Hebei Medical University, Shijiazhuang City, Hebei Province, China; b Department of General Surgery, The Third Hospital of Hebei Medical University, Shijiazhuang City, Hebei Province, China; c Department of General Surgery, The Second Hospital of Hebei Medical University, Shijiazhuang City, Hebei Province, China.

**Keywords:** CEA, circulating tumor cells, colorectal cancer, hepatic metastases, prediction model

## Abstract

Colorectal cancer is one of the most common malignant tumors in the world, and about 50% of its advanced patients will have liver metastasis. Preoperative assessment of the risk of liver metastasis in patients with colorectal cancer is of great significance for making individualized treatment plans. Traditional imaging examinations and tumor markers have some limitations in predicting the risk of liver metastasis. Therefore, it is of great clinical value to explore more sensitive and specific predictive indicators for improving early detection and treatment effect. In recent years, circulating tumor cells (CTCs), as a new biomarker, have attracted much attention because of their close relationship with tumor metastasis and prognosis. The purpose of this study is to collect and analyze the data of colorectal cancer patients treated in our hospital, so as to determine the predictive value of circulating tumor cells before operation and related hematological indexes for liver metastasis after radical resection of colorectal cancer, and to establish the corresponding prediction model to provide gastrointestinal surgeons with more accurate identification of high-risk patients and guidance for treatment. A total of 88 patients were included in this study, and 26 of whom developed liver metastasis after colorectal cancer surgery. The possible related factors are included in the single factor logistic regression, and the results are obtained after analysis. Body mass index, carcinoembryonic antigen (CEA), carbohydrate antigen 19-9, tumor marker CA72-4 (CA72-4), cytokeratin-7 (CK-7), CTC count, and neutrophil-to-lymphocyte ratio (*P* < .2) are risk factors for liver metastasis after radical resection of colorectal cancer. Furthermore, the data obtained were included in multivariate regression analysis, and CEA, CA72-4, CK-7, and CTC counts were independent risk factors for liver metastasis after radical resection of colorectal cancer (*P* < .05). This study confirmed that CEA, CA72-4, CK-7, and CTC counts are independent risk factors for liver metastasis after radical resection of colorectal cancer. In addition, the prediction model of this study can help gastrointestinal surgeons accurately identify patients who are prone to liver metastasis after colorectal cancer surgery.

## 1. Introduction

Colorectal cancer (CRC) is one of the most common and fatal malignant tumors in the world.^[1–3]^ In addition, about 50% of the patients in the advanced stage will have liver metastasis, which will significantly affect the therapeutic effect and survival prognosis.^[4–6]^ Liver metastasis is one of the main forms of postoperative recurrence in patients with CRC.^[7,8]^ Therefore, accurate assessment of the risk of preoperative liver metastasis is very important to optimize the treatment plan and improve the prognosis.^[9,10]^ At present, the traditional prediction methods mainly rely on imaging examination (such as computed tomography scan and magnetic resonance imaging) and some serum tumor markers (such as carcinoembryonic antigen [CEA]). Although these methods are widely used in clinics, they have some limitations in the early identification of micrometastasis or prediction of metastasis risk, which may lead to delayed treatment opportunities.^[11–14]^ In recent years, the study of circulating tumor cells (CTCs) has attracted extensive attention. CTCs are tumor cells that shed into peripheral blood circulation, and their quantity and characteristics are closely related to tumor progression, metastasis, and prognosis.^[15–17]^ Studies have shown that the preoperative level of CTCs is significantly related to the invasion and metastasis risk of CRC.^[18–20]^ Therefore, CTCs may become a powerful tool to predict liver metastasis after CRC surgery. In addition, hematological indexes, such as hemoglobin, white blood cell count, and platelet count, have also been found to be related to the existence and progress of tumors.^[21–23]^ These indicators are usually used to evaluate systemic inflammatory response and nutritional status, and their abnormal changes may reflect the biological behavior of tumors. To sum up, it is expected to improve the accuracy and early identification ability of predicting liver metastasis after CRC surgery by combining preoperative CTCs with hematological indexes for comprehensive evaluation. The purpose of this study is to explore the potential value of preoperative CTCs combined with hematological indicators in predicting liver metastasis after radical resection of CRC and, on this basis, to suggest a prediction model, so as to provide a new risk assessment tool for clinical practice and help formulate a more accurate individualized treatment plan.

## 2. Materials and methods

### 2.1. Research objects

The subjects of this study are patients who visited our hospital from January 2022 to March 2024 and were diagnosed with CRC and accepted radical resection of CRC. There were 88 patients, including 59 males and 29 females, ranging in age from 30 to 88 years. Written informed consent was obtained from the individual for the publication of any potentially identifiable images or data included in this article. The research was approved by the Ethics Committee of The Third Hospital of Hebei Medical University. The number is W-2024-03-1.

### 2.2. Research method

A total of 88 patients were collected in this study. After half a year’s follow-up, 26 patients developed liver metastasis after CRC surgery. The related data and medical history of patients during hospitalization were collected, respectively, to study the related influencing factors of liver metastasis after radical resection of CRC.

### 2.3. Collect indicators

This study collected the patient’s gender, age, body mass index (BMI), and related past medical history. At the same time, relevant hematological indexes, such as albumin and neutrophil-to-lymphocyte ratio (NLR), were collected. The results of tumor markers, serum CTC count, and immunohistochemical results were collected.

### 2.4. Statistical method

In this study, SPSS 25.0 was used for data processing and statistical analysis. The quantitative data conforming to the normal distribution are expressed by the mean ± standard deviation. The differences between groups were analyzed by independent sample *t* test. Comparison between groups that do not obey normal distribution uses a nonparametric test. The qualitative data are expressed by the number and percentage of cases, and the chi-square test is used to determine whether there are differences between groups. First, based on the postoperative liver metastasis of CRC and various clinically related indicators, the analysis was carried out. Then, based on the single factor logistic regression analysis of the collected data, the potential risk factors of liver metastasis after radical resection of CRC were determined. In univariate analysis, the exposure factor with *P* ≤ .2 was selected^[24,25]^ and put into multivariate analysis. The independent risk factors of liver metastasis after radical resection of CRC were obtained. *P* < .05 is statistically significant.

## 3. Results

### 3.1. Assignment table of related indicators in this study

### 3.2. Single factor analysis

A total of 88 patients were included in this study, of which 26 patients developed liver metastasis after radical resection of CRC within half a year (see Table 1). The possible related factors were included in univariate logistic regression, including gender, age, BMI, history of hypertension, history of coronary heart disease, history of diabetes, bad habits of smoking and drinking, tumor type, serum CTC count, serum albumin, NLR, ferritin, CEA, carbohydrate antigen 19-9 (CA199), carbohydrate antigen 125 (CA125), tumor marker CA72-4 (CA72-4), and cytokeratin-7 (CK-7). The results showed that BMI, CEA, CA199, CA72-4, CK-7, CTC count, and NLR were the risk factors for liver metastasis after radical resection of CRC, *P* < .2 (see Table 2).

**Table 1:** Assignment table of related indicators in this study.

Name	Variable assignment and description
Metastases	0 = no, 1 = yes
CK-7	0 = negative, 1 = positive
Hypertension	0 = no, 1 = yes
Coronary heart disease	0 = no, 1 = yes
Diabetes	0 = no, 1 = yes
Smoking	0 = no, 1 = yes
Drinking	0 = no, 1 = yes
Hypoproteinemia	0 = no, 1 = yes
CEA	<5.2 is 0, >5.2 is 1
Ferritin	<400 is 0, >5.2 is 1
CA199	<27 is 0, >5.2 is 1
CA125	<35 is 0, >5.2 is 1
CA72-4	<6.9 is 0, >5.2 is 1

CA125 = carbohydrate antigen 125, CA199 = carbohydrate antigen 19-9, CA72-4 = tumor marker CA72-4, CEA = carcinoembryonic antigen, CK-7 = cytokeratin-7.

**Table 2:** Univariate logistic regression results.

Variables	β	SE	Z	P	OR (95% CI)
Tumor type					
Rectal cancer					1.00 (Reference)
Carcinoma of colon	0.06	0.48	0.12	.904	1.06 (0.41–2.72)
Gender					
Female					1.00 (Reference)
Male	−0.35	0.49	−0.71	.478	0.71 (0.27–1.84)
BMI					
0					1.00 (Reference)
1	−1.37	0.67	−2.04	**.041**	0.25 (0.07–0.95)
History of hypertension					
0					1.00 (Reference)
1	−0.73	0.61	−1.19	.233	0.48 (0.14–1.60)
History of coronary heart disease					
0					1.00 (Reference)
1	−0.05	0.87	−0.06	.953	0.95 (0.17–5.24)
History of diabetes					
0					1.00 (Reference)
1	0.33	0.54	0.61	.544	1.39 (0.48–4.01)
Smoking					
0					1.00 (Reference)
1	−0.56	0.62	−0.91	.364	0.57 (0.17–1.92)
Drinking					
0					1.00 (Reference)
1	−0.40	0.52	−0.78	.437	0.67 (0.24–1.84)
Hypoproteinemia					
0					1.00 (Reference)
1	−1.31	1.09	−1.20	.229	0.27 (0.03–2.28)
CEA					
0					1.00 (Reference)
1	2.63	0.57	4.65	**<.001**	13.89 (4.58–42.09)
Ferritin					
0					1.00 (Reference)
1	18.70	1615.10	0.01	.991	131888918.26 (0.00–Inf)
CA199					
0					1.00 (Reference)
1	1.75	0.56	3.10	**.002**	5.76 (1.91–17.42)
CA125					
0					1.00 (Reference)
1	0.19	0.90	0.21	.833	1.21 (0.21–7.04)
CA72-4					
0					1.00 (Reference)
1	1.44	0.55	2.60	**.009**	4.22 (1.43–12.48)
CK-7					
0					1.00 (Reference)
1	−2.43	0.67	−3.65	**<.001**	0.09 (0.02–0.33)
CTC	0.15	0.04	3.45	**<.001**	1.16 (1.07–1.27)
Age	−0.01	0.02	−0.62	.538	0.99 (0.95–1.02)
NLR	3.24	1.51	2.14	**.032**	25.64 (1.32–498.30)

Bold values indicate *P* < .05.

95% CI = 95% confidence interval, BMI = body mass index, CA125 = carbohydrate antigen 125, CA199 = carbohydrate antigen 19-9, CA72-4 = tumor marker CA72-4, CEA = carcinoembryonic antigen, CK-7 = cytokeratin-7, CTC = circulating tumor cell, NLR = neutrophil-to-lymphocyte ratio, OR = odds ratio, P = *P* value, SE = standard error, β = beta.

### 3.3. Multivariate regression

The 7 risk factors of BMI, CEA, CA199, CA72-4, CK-7, CTC count, and NLR obtained from single factor analysis were further included in multivariate analysis. The results showed that CEA, CA72-4, CK-7, and CTC were independent risk factors for liver metastasis after radical resection of CRC (see Table 3).

**Table 3:** Multivariate logistic regression results.

Variables	β	SE	Z	P	OR (95% CI)
CEA	2.74	0.91	3.00	**.003**	15.48 (2.58–92.89)
CA72-4	2.31	1.04	2.22	**.027**	10.05 (1.31–77.26)
CK-7	−3.94	1.51	−2.61	**.009**	0.02 (0.00–0.37)
CTC	0.20	0.09	2.34	**.019**	1.22 (1.03–1.44)

Bold values indicate *P* < .05.

95% CI = 95% confidence interval, CA72-4 = tumor marker CA72-4, CEA = carcinoembryonic antigen, CK-7 = cytokeratin-7, CTC = circulating tumor cell, OR = odds ratio, P = *P*-value, S.E = standard error, β = beta.

### 3.4. Drawing of nomogram

**Figure 1.**
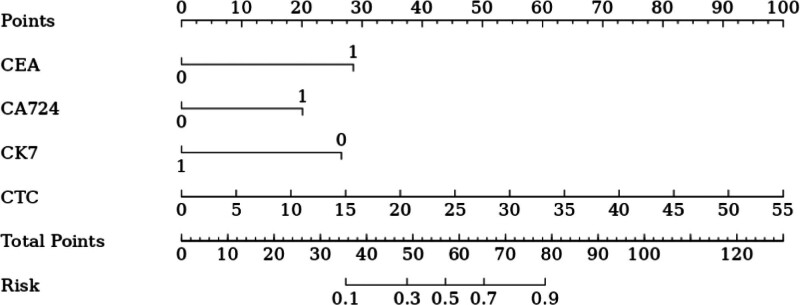
Nomogram prediction of liver metastasis after radical resection of colorectal cancer. CA72-4 = tumor marker CA72-4, CEA = carcinoembryonic antigen, CK-7 = cytokeratin-7, CTC = circulating tumor cell.

**Figure 2.**
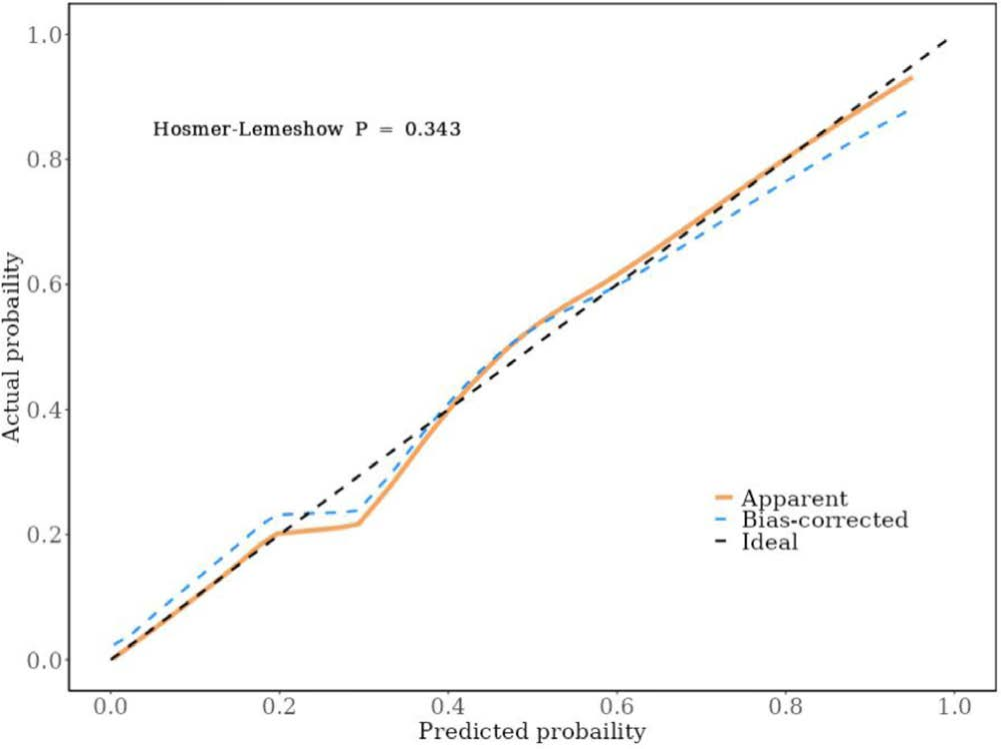
Internal verification of nomogram: calibration curve.

**Figure 3.**
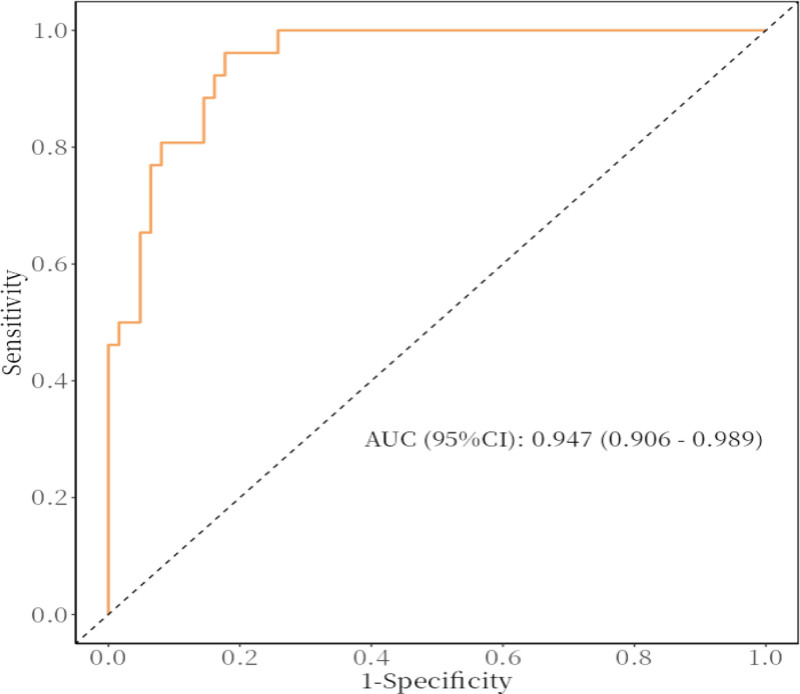
Receiver operating characteristic curve. AUC = area under the curve, CI = confidence interval.

**Figure 4.**
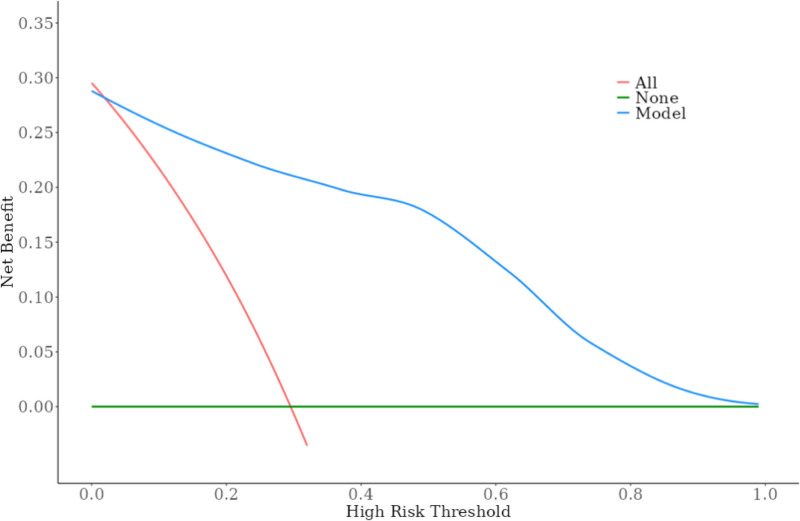
Decision curve in nomogram model.

Based on 7 independent predictors tested by multivariate logistic regression analysis, the risk nomogram of liver metastasis after radical resection of CRC was constructed (see Figure 1). Assign a Nomo score to each independent risk factor. Sum up the total score based on the patient’s clinical characteristics, then locate it on the total points axis. The value on the vertically downward corresponding risk axis is the probability of liver metastasis after radical resection of CRC. The score of each independent predictor corresponds to the upper limit of the score of each independent predictor. The total score of each subject is the sum of the scores of independent predictors. Liver metastasis after radical resection of CRC is determined by the total score on the risk axis of liver metastasis after radical resection of CRC. Subsequently, the model is verified internally, and the bootstrap method in R software is used to repeat sampling 1000 times to verify the nomogram. The calibration curve is close to the ideal curve, which shows that the nomogram predicts the incidence of liver metastasis after radical resection of CRC with a high degree of coincidence with the actual incidence, which shows good prediction performance (see Figure 2). The receiver operating characteristic curve of the nomogram has an area under the curve of 0.947 (95% confidence interval = 0.906–0.989) (see Figure 3). It shows that the nomogram has good discrimination for the high-risk population of liver metastasis after radical resection of CRC. The decision curve of the nomogram shows that when the threshold probability of individuals is >0.05, the model provides more net benefits than the strategy of “everyone intervenes” or “no one intervenes.” This conclusion shows that the nomogram model has good clinical application value in predicting liver metastasis after radical resection of CRC (see Figure 4).

## 4. Discussion

### 4.1. Predictive value of CTC combined with hematological indexes for liver metastasis after radical resection of CRC

In this study, 88 patients with CRC after radical operation were followed up for half a year, of which 26 patients had postoperative liver metastasis. Through univariate logistic regression analysis, the study included many potential factors, including gender, age, BMI, history of hypertension, history of coronary heart disease, history of diabetes, bad habits of smoking and alcohol, tumor type, serum CTC count, serum albumin, NLR, ferritin, CEA, CA199, CA125, CA72-4, and CK-7. The results showed that BMI, CEA, CA199, CA72-4, CK-7, CTC count, and NLR were significantly correlated with postoperative liver metastasis of CRC (*P* < .2), suggesting that these factors may have some influence on postoperative liver metastasis of CRC. Based on single factor analysis, BMI, CEA, CA199, CA72-4, CK-7, CTC count, and NLR were included in multivariate logistic regression analysis. The results showed that CEA, CA72-4, CK-7, and CTC were independent risk factors for liver metastasis after radical resection of CRC. Other factors failed to remain significant in multivariate analysis, which further confirmed the independent predictive value of these variables. The results of this study confirmed that CTCs showed significant predictive value in patients with CRC. CTC count, as an independent risk factor, was included in the final prediction model together with CEA, CA72-4, and CK-7. This finding highlights the role of CTC count in predicting liver metastasis of CRC after operation. The accuracy and clinical application value of CTC count has been verified in this study. By combining with other predictors, high-risk patients can be identified more effectively, and follow-up strategies and intervention measures can be optimized. At present, many studies at home and abroad show that the preoperative CTC level is closely related to the risk of liver metastasis after CRC surgery.^[26–29]^ The existence of CTC not only reflects the metastatic potential of a tumor but also may mark the tiny metastatic focus of a tumor. The author’s team considers that the existence of CTC is closely related to the invasive characteristics of tumors in terms of specific mechanisms. Tumor cells can shed from the primary tumor tissue and enter the blood circulation by acquiring the ability of invasion and migration.^[30,31]^ This process is usually accompanied by changes in adhesion molecules on the surface of tumor cells, such as the down-regulation of E-cadherin, which makes it easier for tumor cells to leave the primary site and enter the bloodstream. Second, the detection of CTC is helpful to reveal the blood flow characteristics of tumor cells, which spread through the blood system and may form tiny metastatic lesions far from the primary lesions. Tumor cells can not only travel far through blood flow but also maintain their survival through interaction with vascular endothelial cells, formation of microthrombosis, and interaction with immune system cells. Together, these factors enable CTC to find and colonize new sites in vivo, thus forming tiny metastatic lesions. This mechanism explains why the detection of CTC can provide important information for the prediction of liver metastasis after radical resection of CRC and provide a valuable basis for the formulation of individualized treatment strategies. In addition, according to Zhao L and Zeng’s research, the high level of CTC before operation significantly indicates the high risk of postoperative recurrence and distant metastasis.^[32,33]^ The detection of CTC is of great value in the early detection of potential micrometastasis and monitoring of tumor burden, which helps clinicians to formulate individualized treatment plans and take timely intervention measures to reduce the risk of postoperative liver metastasis.

### 4.2. Clinical significance of the established model

This study confirmed that CEA, CA72-4, CK-7, and CTC counts are independent risk factors for liver metastasis after radical resection of CRC. By constructing and verifying the nomogram model, the model integrates CTC count and other independent risk factors, showing excellent prediction performance. Its high area under the curve value and good calibration curve show that the model has good applicability and reliability in clinical practice. It is of great clinical significance to establish a prediction model based on preoperative CTC and hematological indexes. This model can comprehensively consider multiple factors and improve the prediction accuracy of liver metastasis after radical resection of CRC. According to the latest research, combining CTC with hematological indicators (such as white blood cell count and hemoglobin level) can significantly improve the predictive ability of the model, thus helping doctors to identify high-risk patients earlier.^[34–36]^ On this basis, this study conducted a short-term follow-up study, and on this basis, identified the independent risk factors of liver metastasis after radical resection of CRC and established a prediction model. Such a model not only helps to optimize the postoperative management of patients but also provides a scientific basis for individualized treatment decisions and further improves the prognosis of patients. In the future, further external verification and large-scale clinical application research will help to verify the stability and universality of the model and promote its application in the postoperative management of CRC.

### 4.3. Why is there such predictive value?

The predictive value of CTC combined with hematological indexes mainly lies in its ability to reflect the biological characteristics and metastatic ability of tumor cells. The existence of CTC indicates that tumor cells have separated from the primary tumor tissue and entered the blood circulation. This process is usually accompanied by tumor cells acquiring highly invasive characteristics, including changing cell adhesion molecules and increasing the activity of matrix metalloproteinases. These characteristics enable tumor cells to break through the tissue boundary of the primary focus and enter the bloodstream, thus showing high metastatic potential. Once tumor cells enter the blood circulation, they can spread to places far away from the primary lesions through the blood flow. It is found that the high level of CTC is usually associated with poor prognosis, because these cells have strong metastatic ability and can colonize in new organ environments and form tiny metastatic lesions. For example, CTC can form tiny metastases in the liver, lung, and other places far away from the primary tumor.^[37,38]^ Sometimes these tiny metastatic lesions may not cause obvious symptoms immediately, but they may lead to disease progression over time. In addition, the detection of CTC is usually combined with the changes in hematological indexes, which often reflect the overall impact of systemic inflammatory reaction or tumor on the host, which further supplements the role of CTC in prediction. Systemic inflammatory reactions, such as the change in white blood cell count and the increase of acute reactive protein level, can indirectly reflect the biological behavior of a tumor and its influence on the host. By comprehensively considering these hematological indexes, the prognosis risk and treatment needs of patients can be more comprehensively evaluated. Therefore, the combination of CTC and hematological indexes can provide a more comprehensive prognosis evaluation. This comprehensive evaluation can reveal the invasion, metastasis potential, and systemic inflammatory response of tumors, thus providing more accurate prognosis information and individualized treatment strategies for clinics.

### 4.4. Optimization direction of future model

Future model optimization can be carried out in the following aspects: combining more extensive clinical data and molecular biological information, such as genome data and protein group data, to enhance the predictive ability of the model. This multi-level data integration can help identify more potential prognostic markers, thus improving the accuracy and reliability of the model. The dynamic changes of CTC were included in the model, and the changing trend of CTC level during postoperative follow-up was considered. Dynamic monitoring can reflect the progress of the disease more accurately, thus optimizing the prognosis evaluation and treatment strategy. Apply advanced machine learning algorithms, such as deep learning and ensemble learning, to improve the performance of the prediction model. Machine learning technology can process a large number of complex data and mine potential patterns hidden in the data, thus improving the prediction accuracy of the model. Carry out large-scale verification in different populations and clinical settings to ensure the universality and practicability of the model. Clinical verification can ensure the adaptability of the model in different environments and help to adjust the model parameters to meet the actual needs.

### 4.5. Limitations

Although this study has practical clinical significance, it does have some limitations. The sample size of this study may not be enough to cover all possible subtypes or stages of CRC, which limits the universal applicability of the results. Future research should consider larger samples and wider patient groups to verify the universality of the research results. In addition, different laboratories and detection methods may lead to differences in CTC counting results, which may affect the reliability of the prediction model. Second, there are some other interfering factors; for example, other health conditions of patients or treatment factors (chemotherapy and other treatment measures) may affect the accuracy of CTC counting. In addition, this study mainly focuses on the risk of liver metastasis in the short term after operation and has not discussed the predictive value of CTC count in long-term follow-up. Long-term research may reveal more about the role of CTC counting in long-term risk transfer.

## 5. Conclusions

This study confirmed that CEA, CA72-4, CK-7, and CTC counts are independent risk factors for liver metastasis after radical resection of CRC. In addition, the prediction model of this study can help gastrointestinal surgeons accurately identify patients who are prone to liver metastasis after CRC surgery.

## Acknowledgements

The authors thank the researchers and study participants for their contributions. In addition, they thank the medical staff in the Department of Clinical Laboratory and Department of General Surgery of The Third Hospital of Hebei Medical University for the support.

## Author contributions

**Data curation:** Tianyi Zhu, Yunsong Li, Rui Li.

**Writing – review & editing:** Tianyi Zhu, Wentao Zhang.

**Writing – original draft:** Yunsong Li, Jingjing Zhang.
